# Exploring the Potential of *Candida* sp. SW4-6 as a Probiotic for Enhancing Water Quality in Aquaculture

**DOI:** 10.3390/microorganisms13010042

**Published:** 2024-12-29

**Authors:** Jie-Ying Li, Chun-Hung Liu

**Affiliations:** Department of Aquaculture, National Pingtung University of Science and Technology, Pingtung 912, Taiwan; james19970806@icloud.com

**Keywords:** *Candida* sp., nitrite, carbon source, C/N ratio, water quality, environmental condition

## Abstract

Aquaculture, a vital industry supplying a significant portion of the world’s seafood, faces challenges such as the deterioration of the aquaculture environment. The objective of this study was to isolate and identify microorganisms with the capacity to eliminate nitrite in water from shrimp ponds and evaluate their potential as probiotics to improve water quality. Additionally, the study also determines the ideal conditions for the probiotic to effectively reduce nitrite-N and ammonia-N. Water samples were collected from four shrimp ponds (SW1, SW2, SW3, SW4) and isolates were obtained. Among all the samples, SW4 was the most effective in reducing the concentration of nitrite-N. Upon further isolation of SW4, the strain SW4-W6 showed significant nitrite-N reduction capabilities compared to the 19 other isolates tested. Through morphological, genetic (ITS sequence), and phylogenetic analyses, strain SW4-6 was identified as *Candida* sp. The isolation of *Candida* sp. SW4-6 showed superior nitrite-N and ammonia-N reduction capabilities, with sucrose as the carbon source and complete reduction observed at a C/N ratio of 15–20. Gene expression analysis revealed the up-regulation of nitrite reductase in SW4-6 after inoculation, with significantly higher expression observed with sucrose as the carbon source. Salinity and temperature significantly influenced nitrite-N and ammonia-N reduction by SW4-6, with higher temperatures (30 °C) and 0% NaCl favoring faster reduction rates. *Candida* sp. SW4-6 emerges as a promising probiotic candidate for aquaculture water quality management due to its efficient nitrite-N and ammonia-N reduction capabilities under optimal conditions. Its virulence profile and ability to thrive across various salinity and temperature conditions further support its potential applicability in aquaculture.

## 1. Introduction

The rapid growth of the global population poses a significant challenge to ensuring an adequate food supply for the estimated 9 billion people expected by 2050. Aquaculture is considered a pivotal contributor to meeting the dietary demands of all individuals. According to the Food and Agriculture Organization (FAO), capture fishery has remained stagnant since the mid-1990s, while aquaculture’s annual growth rate has increased. In 2022, global aquatic animal production reached 185.4 million tons, with aquaculture contributing approximately 94.4 million tons (51% of the total), surpassing capture fisheries, which accounted for 91 million tons (49%) for the first time [1]. Among aquaculture species, the white shrimp, *Penaeus vannamei*, has emerged as the most highly produced species, with an output of 6.8 million tons in 2022 [1]. This trend signifies a significant shift in seafood sources, with aquaculture currently being the predominant source.

With an increasing emphasis on sustainability and food security, meeting the growing demand for seafood requires the scaling up of production. However, this presents some challenges, such as a higher stocking density in ponds, which can result in the accumulation of significant amounts of organic matter. This accumulation is accompanied by the production of elevated levels of ammonia-N and nitrite-N, both of which can have detrimental effects on the survival and growth of aquaculture animals [2,3]. Traditionally, the most common approach to mitigating harmful substances in aquaculture systems is water change. This method allows for the direct control of toxic metabolites like ammonia-N and nitrite-N, keeping their concentrations low. However, this method releases a significant amount of aquaculture wastewater into the natural ecosystem, leading to eutrophication and potentially facilitating the spread of pathogens [4,5]. For instance, Kang et al. [4] found that an increased diatom abundance correlated with a rise in heterotrophic/mixotrophic cysts, suggesting that excess uneaten food and fish farm waste contribute to eutrophication in Korea’s southern coastal waters. Additionally, infectious hypodermal and hematopoietic necrosis virus (IHHNV) has been introduced to wild penaeid shrimp populations in the Gulf of California, likely following the importation of *P. vannamei* postlarvae to local shrimp farms, where it spread to Pacific blue shrimp, *P. stylirostris,* and possibly other native species [5].

Nitrification is a process performed to remove ammonia and nitrite in aquaculture systems by ammonia-oxidizing bacteria and subsequent nitrite-oxidizing bacteria [6,7]. However, the nitrification process carried out by autophytic bacteria is very slow and requires a surface for attachment to function or propagate. To support this process, biofilters are an essential component in recirculating aquaculture systems [8]. Several types of biofilters have been developed, including bio-scrubbers, trickling filters, and slow sand filters. These systems immobilize microorganisms, including nitrifying bacteria, on solid substrates to facilitate the degradation of pollutants. In contrast to nitrification, heterotrophic ammonia assimilation can effectively remove ammonia and convert it to a bacterial biomass such as glutamate and glutamine, the major amino group donors for all nitrogen-containing compounds, such as other amino acids, purines, pyrimidines, and vitamins [9]. It has been observed that certain bacteria possess a unique capacity for heterotrophic ammonia assimilation, which can have significant implications for improving aquaculture systems. These bacteria, including, but not limited to, *Bacillus* spp. [10], *Zobellella* [11], *Pseudomonas* [12], and *Acinetobacter* [13], have been shown to effectively reduce ammonia-N levels in water, thus making them a potential solution to maintaining a healthy aquatic environment.

Nitrite is another nitrogen metabolite that can also accumulate in water and pose a toxic threat to aquaculture animals. The process of dissimilatory nitrite reduction is mediated by nitrite reductase, with cytochrome c-type nitrite reductase being recognized as the dissimilatory ammonia-forming nitrite reductase [14]. Despite the widespread existence of nitrite reductase microorganisms, nitrite is often found to accumulate at higher levels in aquaculture systems. Therefore, the non-pathogenic bacteria that use nitrite as a nitrogen source and are able to reduce the levels of ammonium in water can be selected as a probiotic for water treatment in aquaculture.

*Candida* are yeast species known for their potential use in wastewater treatment processes [15]. Yeasts, as biodegrading microorganisms, exhibit strong environmental adaptability and resistance to acidity, tolerance to concentrated organic substrates, and the rapid degradation of organic matter [16]. Several studies have reported the treatment of industrial, municipal and agricultural wastewaters using yeast species, including *Candida* spp. [17]. *Candida maltosa* has been shown to degrade phenol up to 1700 mg L^−1^ [18]. The yeast isolates *C. halophila* and *Rhodotorula glutinis* effectively achieved an 85% reduction in the chemical oxygen demand of glutamate fermentation wastewater [19]. Similarly, a strain of *C. utilis* that utilizes ammonia and grows well with free ammonia below 197 mg L^–1^ was also isolated by Ding et al. [20]. These promising capabilities highlight the potential application of *Candida* spp. in aquaculture systems for the removal of nitrogen and other pollutants. Such pollutants can be toxic to aquaculture species and may impair growth performance [2,21]. The use of *Candida* spp. could, therefore, contribute to maintaining healthier aquaculture environments and enhance productivity.

The carbon-to-nitrogen (C/N) ratio is pivotal in composting, as microorganisms need a balanced ratio of carbon to nitrogen to sustain their activity [22]. Considering the crucial role of the C/N ratio, understanding its impact on microbial nitrogen utilization becomes essential, especially when employing the microorganism as a probiotic for water treatment in aquaculture.

In order to establish an eco-friendly approach to improving the condition of water in aquaculture, this study aims to isolate microorganisms capable of reducing ammonia and nitrite in aquaculture systems. The efficiency of ammonia and nitrite removal was also evaluated under different C/N rations. The results of this study could contribute to the development of probiotics for enhancing the water quality in aquaculture. The isolated microorganisms, if successfully used as probiotics, could provide a sustainable and cost-effective solution for managing water quality in aquaculture systems.

## 2. Materials and Methods

### 2.1. Isolation

This screening was conducted in cement tanks situated at the aquaculture farm of the National Pingtung University of Science and Technology (NPUST), Pingtung, Taiwan, in July 2021. The white shrimp cultured in these tanks had been maintained for over 2 months, with an average weight of 8.25 ± 2.87 g, during which the water quality was carefully monitored and remained stable. This consistent and controlled environment provided an optimal setting for isolating potential probiotics.

To determine the microorganism’s ability to reduce nitrite, water samples (20 psu) were collected from four different white shrimp, *P. vannamei,* tanks (SW1, SW2, SW3 and SW4) and stored in 50 mL centrifuge tubes. The samples were allowed to stand for 15 min, and then the supernatant from the water samples was inoculated in a 250 mL Erlenmeyer flask containing N medium, with nitrite-N as the sole nitrogen source (1 g of glucose, 2 g of KH_2_PO_4_, 0.05 g of MgSO_4_·7H_2_O, 0.02 g of FeSO_4_·7H_2_O, 20 g of NaCl and 20 mL of filter-sterilized 280 mg L^−1^ NO_2_-N solution in 1 L of water, pH 7.0); this was cultured in an orbital shaking incubator at 30 °C and 150 rpm. To confirm the presence of microorganisms capable of reducing the nitrite in the samples, the concentration of nitrite-N in the medium was assessed at 0, 24, 48, 72, 96 and 120 h following the methods described by Bendschreider and Robinson [23]. All experiments were performed in triplicate. The sample exhibiting the greatest nitrite-N reduction capabilities in the N medium was chosen for subsequent isolation. The microorganisms were spread on the surface of a solid N medium (prepared with the aforementioned recipe with 1.5% agar) and cultured in an incubator at 30 °C for 48 h. Subsequently, 20 colonies were randomly chosen, inoculated in N medium, and then cultured in a shaking incubator at 30 °C and 150 rpm. The nitrite-N concentration was analyzed at 0, 24, 48 and 72 h to identify the strain with the greatest nitrite-N reduction capabilities.

### 2.2. Microorganism Morphology and Growth

To characterize the microorganism, the Gram-staining technique was used, and the morphological features were observed under a light microscope. The growth tolerance in different saline and temperature conditions was assessed on tryptic soy agar (TSA, Difco, MD, USA) supplemented with NaCl concentrations and temperatures ranging from 0.5 to 12% and 0 to 50 °C, respectively.

### 2.3. Identification

The strain with the best nitrite-N reduction capabilites was identified by Tri-Biotech, Inc. (Taipei, Taiwan) using internal transcribed spacer sequencing (ITS) with the following forward primers: 5′-ACAAGGTTTCCGTAG-3′ and revers: 5′-ATGCTTAAGTTCAGC-3′. The ITS sequence was compared to known sequences in GenBank using the Basic Local Alignment Search Tool (BLAST) (NCBI Nucleotide BLAST. https://blast.ncbi.nlm.nih.gov/Blast.cgi, accessed on 2 February 2024). Subsequently, the phylogenetic tree was constructed using molecular evolutionary genetics analysis (MEGA) 4.1 software (available online: http://www.megasoftware.net/, accessed on 2 February 2024). The tree was constructed using the neighbor-joining method. The values for each internal branch represent the percentage of bootstrap obtained from 1000 replicates. The tree was drawn to scale, with equivalent branch lengths and evolutionary distances.

### 2.4. Virulence Assessment for Aquatic Animals

Several aquatic species, including ten fish, four crustaceans and two bivalves (Table 1), were exposed to the microorganism for 7 d to evaluate the safeness of the isolated strain. The test animals were sourced from private farms, then transferred to the aquafarm at NPUST, Taiwan, and acclimatized for 7 d. During the acclimation period, fish and shrimp were fed with a commercial diet twice daily, while clams were fed with *Isochrysis galbana* twice daily. The assessment was carried out in aquaria tanks containing 20 L of water. Each species was evaluated in triplicate, with each replicate consisting of 10 animals.

The virulence assessment was conducted by immersing the animals in water containing 10^6^ colony-forming unit (cfu) mL^−1^ for 7 d. Throughout the trial, water was renewed daily while maintaining the microorganism concentration. The death rate of animals exposed to the microorganism was observed twice daily, once at 9:00 am and again at 16:00 pm. During the experiment, the water temperature was maintained at 28 °C using heaters, while the dissolved oxygen (DO) levels were sustained at ≥5 mg L^−1^ through continuous aeration.

All procedures in this study were conducted in accordance with the National Pingtung University of Science and Technology Affidavit of approval of Animal Use Protocol (IACUC Approval No.: 110–138).

### 2.5. Carbon-to-Nitrogen (C/N) Ratio for Nitrite-N and Ammonia-N Reduction Measurement

To ensure that the C/N ratio was optimal to support nitrite-N and ammonia-N reduction in water, different mediums with C/N ratios of 10, 15 and 20 were prepared using glucose (Merck, Boston, MA, USA) or sucrose (SIGMA, Saint Louis, MO, USA). A detailed description of the medium components used in the experiment is presented in Supplementary Table S1. The initial concentrations of nitrite-N and ammonia-N were set at 5 mg L^−1^ using sodium nitrite and ammonium chloride, respectively. The mediums were inoculated 10^4^ cfu mL^−1^ with b microorganism and cultured under the controlled conditions of 30 °C and 150 rpm in an incubator. The concentrations of nitrite-N and ammonia-N were measured at the beginning of the study and at, 6, 12, 24, 48, and 72 h.

### 2.6. Gene Expression of Nitrite Reductase at Different C/N Ratios

Culture media with different C/N ratios were prepared using sodium nitrite as a nitrogen source, as described in Section 2.5. These media were utilized to conduct a gene expression analysis of nitrite reductase through SYBR Green real-time PCR at the start of the experiment and at 6, 12, 24, 48, and 72 h. Each experimental group was conducted in triplicate.

Microorganisms were harvested via centrifugation at 3000× *g* for 10 min at 4 °C, washed twice with PBS and then underwent total RNA extraction using the REzol™ C & T reagent (AMRESCO, Solon, OH, USA). After that, first-strand complementary DNA (cDNA) was synthesized using SuperScript^TM^ II Reverse Transcriptase (Invitrogen, Carlsbad, CA, USA) with oligo-d (T)_18_ primer. The reaction was performed according to the manufacturer’s instructions. To enhance the specificity and accuracy of the analysis of nitrite reductase expression, primers were designed specifically based on the *Candida* sp. nitrite reductase sequence (accession number: PP759302) using Primer Express (Primer Express™ Software v3.0.1, Applied Biosystems, Foster, CA, USA). The primers used were Q-CpW6 YNI-F: 5′-ATTGGACGAAAAAGGTGGAAAC-3′ and Q-CpW6 YNI-R: 5′-TCTTGCTGTCACATTCAAGTCTGTAA-3′. To normalize the expression of the target gene across different treatments, 18S rDNA of *Candida* sp. was employed as an internal control gene. The primers used for the amplification of 18S rDNA were Q-18S rDNA-F: 5′-GAGAAACGGCTACCACATCCA-3′ and Q-18S rDNA-R: 5′-CGTGTCGGGATTGGGTAATT-3′. The reactions were conducted in a 96-well plate and the relative mRNA expression of target genes to the reference gene was calculated using the 2^−ΔΔCt^ method [24]. The results are presented as the mRNA expression relative to the beginning.

### 2.7. Evaluation of Nitrite-N and Ammonia-N Removal Efficiency by Probiotic at Different Salinity and Temperature

The media containing sodium nitrite or ammonium chloride as the sole nitrogen source were prepared as described in Section 2.5 to analyze the removal efficiency of nitrite-N and ammonia-N by the isolate under varying environmental conditions. The initial concentrations of nitrite-N and ammonia-N were both 5 mg L^−1^, and the C/N ratio was set at 15. Tests for the elimination of nitrite-N and ammonia-N were conducted in media supplemented with 0% and 3.5% NaCl. Following inoculation, the media were placed in incubators at 20, 25 and 30 °C, and the nitrite-N and ammonia-N levels were measured after 0, 6, 12, 24, 48 and 72 h. Each treatment was performed in triplicate.

### 2.8. Utilization of SW4-6 to Improve Water Quality in Shrimp Aquaculture

Strain SW4-6 was cultured as aforementioned. The pellet was then harvested by centrifugation for 20 min at 2840× *g* and 4 °C, resuspended in 100 mL of 10% skim milk and stored at −80 °C. The frozen sample was lyophilized using a freeze-dryer, homogenized, and then diluted with maltodextrin as excipient to a final concentration of 10^8^ cfu kg^−1^. The resulting powder was stored at 4 °C until use.

To assess the feasibility of probiotic application in aquaculture for water quality improvement, six separate outdoor cement tanks (no. 1 to 6), each containing 300 tons of water at 25 psu, were used for this experiment. At the start of the trial, the ponds were stocked with white shrimp that had been raised for 2 months at an initial density of 200 shrimp per ton. The concentrations of ammonia-N and nitrite-N in each pond were measured as previously described. The concentrations of ammonia-N and nitrite-N in ponds no. 1, no. 2, no. 3, no. 4, no. 5 and no. 6 were 0.57 mg L^−1^ and 0.67 mg L^−1^, 0.69 mg L^−1^ and 0.84 mg L^−1^, 0.89 mg L^−1^ and 1.11 mg L^−1^, 0.77 mg L^−1^ and 1.56 mg L^−1^, 0.79 mg L^−1^ and 1.63 mg L^−1^, and 1.13 mg L^−1^ and 0.77 mg L^−1^, respectively. The first three tanks served as the control, without the addition of sugar and probiotics. The latter three ponds were treated with sugar (sucrose from the Taiwan Sugar Corporation, Tainan, Taiwan) to achieve a C/N ratio of 15 based on the concentrations of ammonia-N and nitrite-N, along with the addition of the probiotic (300 g of probiotic powder in each tank). Thereafter, the concentrations of ammonia-N and nitrite-N were assessed according to the aforementioned methods at 12, 24, 48 and 72 h, respectively. The efficiency of ammonia-N or nitrite-N degradation by the probiotic was expressed as the percentage reduction in the concentrations of ammonia-N or nitrite-N in water.

### 2.9. Statistical Analysis

The experimental data were subjected to statistical analysis using one-way ANOVA and multiple-way ANOVA to identify differences among groups. Before conducting the ANOVA, Levene’s test and the Shapiro–Wilk test were applied to evaluate the homogeneity of variance and data normality. A post hoc multiple comparisons (Duncan’s) test was conducted to examine significant differences among treatments. SPSS (IBM SPSS Statistics for Windows, Version 22.0., IBM Corp., Armonk, NY, USA) was utilized for the statistical analysis in this study. Statistically significant differences were considered when *p* < 0.05.

## 3. Results

### 3.1. Microorganism Isolation and Their Ability to Reduce the Level of Nitrite

**Figure 1:**
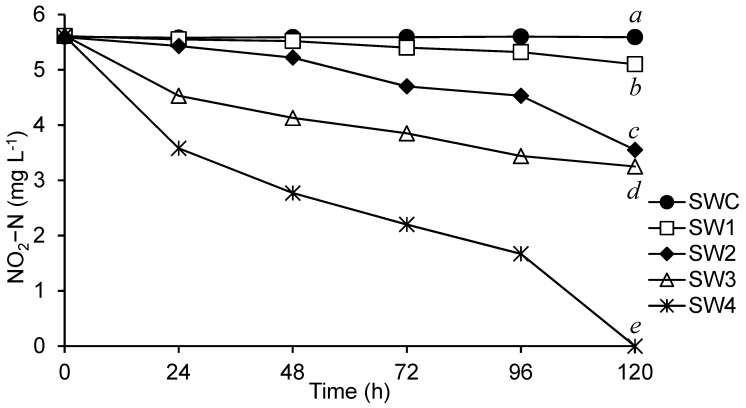
Reduction in nitrite-N in media inoculated with water from four shrimp ponds. SWC: medium without inoculation; SW1~SW4: media inoculated with water from different shrimp ponds. Data are presented as mean ± S.E. Data labeled with different letters at the end of the experiment indicate significant differences among treatments (*p* < 0.05). *n* = 3.

**Figure 2:**
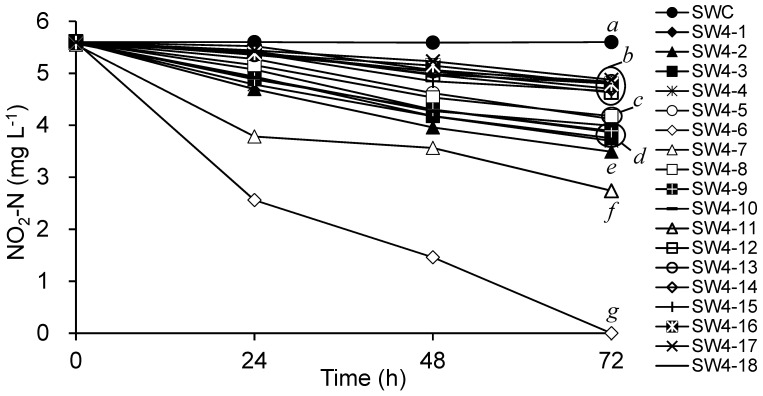
Comparative study of nitrite-N reduction by isolates from SW4 samples. SWC: medium without microorganism inoculation. Data are presented as mean ± S.E. Data labeled with different letters at the end of the experiment indicate significant differences among treatments (*p* < 0.05). *n* = 3.

The water samples collected from the shrimp ponds were inoculated in N medium to screen for microorganisms capable of reducing nitrite. Potential colonies were observed in the SW1 SW2, SW3, and SW4 groups (Figure 1). No reduction in the nitrite-N concentration was observed in the SWC group. Although all groups showed a decreasing trend, the SW4 group exhibited a more rapid decline in nitrite-N at 120 h compared to the other samples. Therefore, SW4 was chosen for subsequent isolation procedures. The nitrite-N reduction capabilities of 20 isolates derived from SW4 are presented in Figure 2. Nitrite-N reduction was observed in all isolates; however, only SW4-6 completely depleted nitrite-N in medium within 72 h. The strain SW4-6 was selected for further identification.

### 3.2. Microorganism Identification

**Figure 3:**
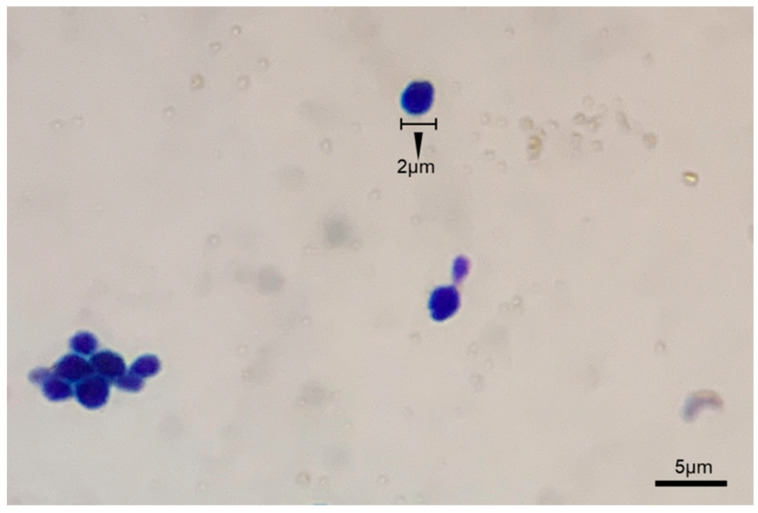
Microscopic image of *Candida* sp. SW4-6 isolated cells displayed for observation with a magnification of 1000×.

**Figure 4:**
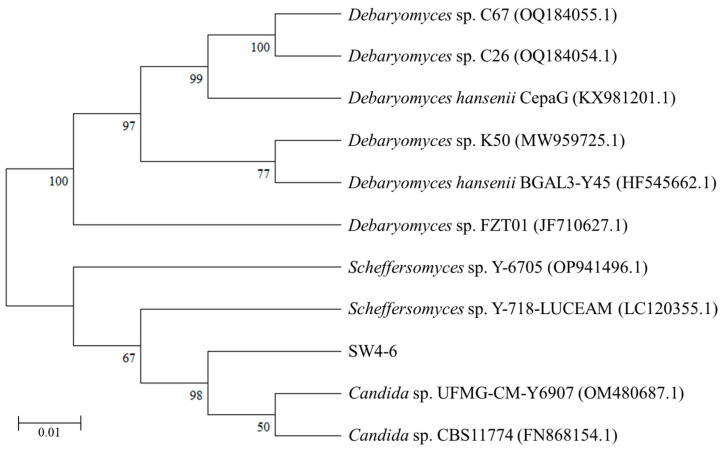
Phylogenetic tree based on ITS sequences of different yeasts.

Following a 2-day incubation period, the SW4-6 isolates displayed round, smooth, and well-defined edges, with a white creamy appearance (Supplementary Figure S1). These colonies grew at temperatures ranging from 20 to 40 °C and demonstrated tolerance to salinity levels within the range of 0.5~12% (Table 2). The size of the isolate was approximately 2 µm, and pseudohyphae were not observed under the microscope (Figure 3). The ITS sequence was employed to identify the nitrite-N reduction capabilities of the SW4-6 isolate. A 624 bp ITS sequence was determined and deposited in GenBank under accession number PP757925. To elucidate the phylogenetic relationships between the SW4-6 isolate and existing isolates, a combined dataset of ITS sequences for the yeasts was selected from the NCBI database to generate a phylogenetic tree (Figure 4). All sequences obtained revealed that the SW4-6 isolate showed 100% and 98.1% similarity to the *Candida* sp. CBS11774 (GenBank accession number: FN868154.1) and *Candida* sp. UFMG-CMY6907 (GenBank accession number: OM480687.1) strains, respectively, which were grouped together in the same clade and indicated a strong supportive bootstrap value. Based on the morphological characteristics, ITS sequence and phylogenetic analysis, it was confirmed that the isolate was *Candida* sp.

### 3.3. Virulence Assessment

In order to ensure the virulence of *Candida* sp. SW4-6, a test was conducted to assess its toxicity to aquaculture animals, including ornamental species, using the immersion method. There were no incidents of mortality and clinical abnormalities observed in any animal exposed to *Candida* sp. SW4-6 during the 7-day experiment (Table 1). These results affirm the virulence of *Candida* sp. SW4-6 and its potential use in aquaculture for improving water quality.

### 3.4. Optimal C/N Ratio for the Reduction of Nitrite-N and Ammonia-N Using Candida sp. SW4-6

Table 3 presents the results of nitrite-N reduction by *Candida* sp. SW4-6 under various carbon sources and C/N ratios. The findings reveal that sucrose is a more efficient carbon source than glucose, as evidenced by the complete reduction in the nitrite-N concentration to zero at 48 h with a C/N ratio of 20. Moreover, no nitrite-N was detected at 72 h in the groups with C/N ratios of 15 and 20 when sucrose was used as the carbon source. In contrast, when glucose was used, nitrite-N was detectable in all C/N ratio groups. The findings indicate that the choice of carbon source, time and C/N ratio significantly impacted nitrite-N reduction.

The ammonia-N concentrations rapidly decreased from approximately 5 mg L^−1^ to zero within 12 h, irrespective of the type of carbon source used (glucose or sucrose) and the C/N ratio (10 and 20) (Table 4). However, the reduction in the ammonia-N concentration was found to be comparatively faster in the sucrose groups. In particular, the concentrations of ammonia-N at C/N ratios of 15 and 20 at 6 h, as well as C/N ratios of 10 at 12 and 24 h, were significantly lower compared to the ammonia-N concentrations at the same C/N ratios when glucose was used as the carbon source (Table 5). These findings also revealed the significant effect of all conditions, including carbon sources, C/N ratios and time intervals.

### 3.5. Gene Expression of Nitrite Reductase at Various Carbon Sources and C/N Ratios

The gene expressions of nitrite reductase were up-regulated after inoculation with *Candida* sp. SW4-6 (Table 5). However, a significantly higher relative gene expression of nitrite reductase was observed in groups that used sucrose at C/N ratios ranging from 10~20, compared to the groups that used glucose. Statistical analysis further revealed that different culture conditions, such as different carbon sources, C/N ratios, and time intervals, were significant factors that affected the gene expression of *Candida* sp. SW4-6.

### 3.6. Salinity and Temperature Influence the Elimination of Nitrite-N and Ammonia-N by Probiotic

Salinity and temperature significantly influenced the ability of the microorganism *Candida* sp. SW4-6 to reduce nitrite-N (Table 6). Interestingly, when sucrose was utilized as the carbon source at a C/N ratio of 15, *Candida* sp. SW4-6 showed a better nitrite-N reduction capacity in the medium without NaCl supplementation compared to the group with 3.5% NaCl. Moreover, an increase in temperature from 20 to 30 °C in both NaCl groups resulted in a significantly faster reduction in nitrite-N. Both salinity and temperature significantly influence nitrite-N reduction by *Candida* sp. SW4-6 (see Supplementary Table S2 for multi-way ANOVA results).

In the absence of NaCl supplementation, *Candida* sp. SW4-6 eliminated ammonia-N significantly faster than in a medium with 3.5% NaCl, similar to the reduction in nitrite-N (Table 7). Media without NaCl supplementation showed no ammonia-N within 24 h, while detectable levels were present in media with 3.5% NaCl after 24 h of inoculation. Temperature was a significant factor, with higher temperatures resulting in faster ammonia-N elimination in both NaCl groups. Moreover, the salinity, temperature, and time interval all had a significant impact on ammonia-N elimination by *Candida* sp. SW4-6, with salinity and temperature having a synergistic effect on ammonia-N reduction (see Supplementary Table S3 for multi-way ANOVA results).

### 3.7. The Practical Application of Probiotics in Shrimp Ponds

**Figure 5:**
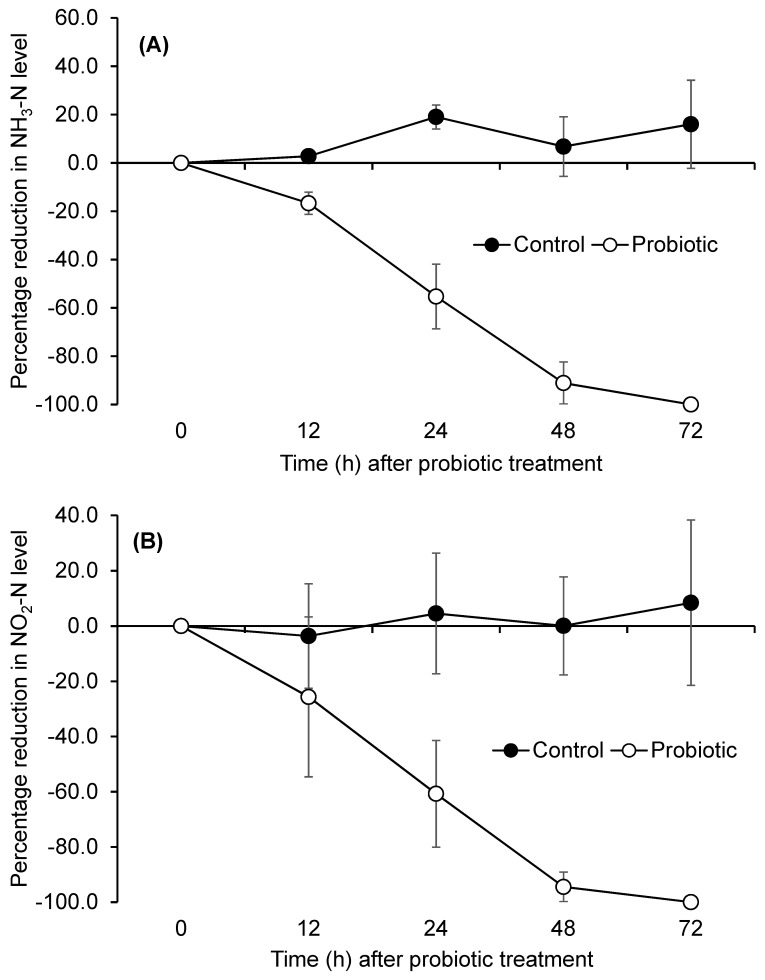
The concentrations of ammonia-N (**A**) and nitrite-N (**B**) in shrimp ponds after probiotic treatment.

The ammonia-N and nitrite-N levels in water were entirely reduced when probiotics were applied to the shrimp ponds at a C/N ratio of 15 (Figure 5A,B). No reduction in ammonia-N and nitrite-N was observed in the control ponds; however, following the addition of the probiotic and sugar, the concentrations of ammonia-N and nitrite-N decreased after 6 h, achieving complete elimination by 72 h of probiotic treatment.

## 4. Discussion

Maintaining optimal water quality levels is crucial for the success of aquaculture. Nitrite, a byproduct of ammonia oxidation, can accumulate to toxic levels in aquaculture systems when the nitrification process becomes impaired [2,21]. To address this issue, the capacity for nitrite reduction was employed as a key criterion for screening potential probiotic candidates. Various methods have been developed to effectively improve water quality, including the use of ozone [25], zeolite [26], biochar [27], microorganisms [28] and others. Among these options, the use of microorganisms for improving pond conditions is deemed more cost-effective, environmentally friendly, and long-lasting [28]. Two bacteria, *Bacillus amyloliquefaciens* and *Pseudomonas stutzeri*, have shown the highest potential degradation of ammonia, while *B. cereus* has been found to degrade nitrite to treat aquaculture wastewater [28]. Three bacteria capable of removing ammonia nitrogen were isolated from carbonate saline–alkali soil and water environments, namely *B. idriensis* CT-WN-B3, *B. australimaris* CT-WL5-10, and *P. oleovorans* CT-WL5-6. These bacteria have demonstrated removal rates of up to 3 × 10^−13^ mg cfu^−1^ h^−1^ [3]. Additionally, an ascomycetous yeast, *Candida* sp. SW4-6, isolated from a shrimp pond, has demonstrated significant potential in removing nitrite-N and ammonia-N, achieving complete removal within 48 h when the initial concentrations of nitrite-N and ammonia-N were approximately 5 mg L^−1^. Consequently, it is believed that *Candida* sp. SW4-6 could be a promising probiotic for enhancing conditions in aquaculture ponds.

The application of yeast for water quality treatment in aquaculture ponds remains an area of limited research. However, yeast species belonging to *Candida* and *Hansenula* genera, as well as species such as *Saccharomyces cerevisiae*, *Pichia anomala*, or *Kluyveromyces marxianus,* have shown effectiveness in removing organic compounds or heavy metals from aqueous media or industrial pollutants [15,29,30,31]. Yun et al. [32] reported the use of marine red yeast, *Rhodosporidiums sphaerocarpum,* for the removal of ammonia from shrimp ponds. Previous investigations have demonstrated that *C. palmioleophila* is proficient in assimilating palm oil [33] and degrading azo dyes [34], indicating its potential for environmental remediation. *C. tropicalis* cells, predominantly employed as biosorbents for heavy metal decontamination due to their resistance, have demonstrated proficiency in both single and binary systems for adsorbing copper (II) and phenols, as well as degrading diesel oil as a biofilm [35]. The yeast *C. tropicalis*, capable of simultaneous nitrogen and phosphorus removal, was isolated, and no nitrite and nitrate were produced in the ammonia removal process; however, gaseous phosphorus compounds were produced in its phosphate degradation process [36]. In this study, the isolate *Candida* sp. SW4-6 demonstrated the ability to grow in a medium with nitrite or ammonium as the sole nitrogen source, revealing its potential for removing nitrite-N and ammonia-N from aquaculture ponds. The successful deployment of this yeast strain could help in maintaining optimal water conditions for aquaculture animals.

Yeast is a ubiquitous microbe that exhibits extremely high adaptability to a wide range of environments, including soil, freshwater, marine environments and the surface of organisms [15]. Generally, yeast is capable of growing within a temperature range of 0 °C to 47 °C and is known to enter a dormant state at low temperatures until favorable conditions arise. However, at temperatures exceeding 50 °C, yeast cells may undergo cellular deterioration, ultimately leading to cell death. Typically, yeast thrives best at temperatures ranging from 25 °C to 30 °C [37]. The results of this study demonstrate that *Candida* sp. SW4-6 can tolerate temperatures up to 40 °C. This observation aligns with findings from other studies on *Candida* [38,39,40]. Furthermore, this strain exhibited a tolerance to high-salt (12% NaCl) environments, which is consistent with the characteristics of other *Candida* species, including *Candida etchellsii* [41]. Most aquaculture animals thrive in temperatures below 30 °C and are not exposed to high salt concentrations, making this yeast strain suitable for practical applications. Although *Candida* sp. SW4-6 might enter a dormant state below 10 °C, few aquaculture species have been cultured in such cool environments.

The isolation of *Candida* sp. SW4-6 has been proposed as a promising approach to improving the water quality of aquaculture ponds. Therefore, ensuring the virulence of this isolate is crucial in determining its suitability as a probiotic for aquaculture purposes. Yeast belongs to the fungi kingdom, and high-density aquaculture conditions can often lead to compromised health in cultured animals, triggering fungal diseases [42,43] such as Saprolegniasis, caused by *Achlya* sp. and *Saprolegnia* sp. [44]. Despite these findings, the virulence test conducted with *Candida* sp. SW4-6 on ten fish species, four crustacean species, and two bivalves showed no animal deaths after immersion for 7 d. Similarly, the injection-based virulence tests conducted on nine fish species and three crustacean species also resulted in no recorded mortalities over 7 days (Supplementary Table S5). These findings collectively indicate that *Candida* sp. SW4-6 is non-pathogenic, non-toxic, and safe for use in aquaculture, supporting its potential as an effective water quality enhancer.

Nutrients are essential for the growth of microorganisms, and a nutritional imbalance can lead to an unstable process performance, which is a major limiting factor. Nitrogen and carbon play crucial roles as key nutrients, with their ratio being deemed essential for ensuring satisfactory microorganism performance [45]. Aquatic animals, such as fish and shrimp, are ammoniotelic [46], whereby ammonia is the principal nitrogenous end product, and nitrogen metabolism may lead to substantial accumulations of ammonia-N and nitrite-N during ineffective nitrification. While nitrogen sources vary in aquaculture ponds, carbon sources often become a limiting factor in the growth of microorganisms [47]. Carbon sources play a crucial role in influencing both the growth of microorganisms and their rates of ammonia-N and nitrite-N degradation in water [48,49]. Thus, understanding the optimal C/N ratio for the microorganisms used in ammonia-N and nitrite-N removal within aquaculture systems is essential. In this investigation, the removal of ammonia-N and nitrite-N by *Candida* sp. SW4-6 was influenced by both the carbon source and the C/N ratio. Sucrose and C/N ratios ranging from 15 to 20 yielded more favorable results. In fact, *Candida* sp. SW4-6 exhibited a significant capacity to reduce ammonia-N and nitrite-N in controlled laboratory conditions, and it also showed considerable effectiveness in mitigating these nitrogen metabolisms in shrimp ponds. Given these findings, this strain isolated from shrimp ponds represents a potential microorganism for improving the water quality in aquaculture systems.

The effectiveness of microorganisms in degrading nitrite-N is closely linked to the presence of nitrite reductase. Studies have indicated that yeast species, including *Hansenula anomala*, have the ability to assimilate nitrite-N [50], and research has delved into the functional structure of nitrite reductase [51]. Consequently, it is suggested that variations in the ability of *Candida* sp. SW4-6 to degrade nitrite-N under different conditions may be attributed to fluctuations in the gene expression of nitrite reductase. The findings of this study illustrate that the gene expression of nitrite reductase is significantly enhanced under high C/N ratios, regardless of the carbon source. Moreover, when sucrose serves as the carbon source, the gene expression of nitrite reductase in *Candida* sp. SW4-6 is higher compared to the glucose-treated group. These results align with the observation that *Candida* sp. SW4-6 exhibits superior nitrite-N degradation capabilities when utilizing sucrose as a carbon source and operating under high C/N ratios.

Sucrose, a naturally occurring and widely consumed dietary sugar, is highly cariogenic and serves as a substrate for the synthesis of extracellular and intracellular polysaccharides within plaque biofilms. Similarly, glucose, a monosaccharide derived from sucrose fermentation, plays a significant role in the development of oral biofilms [52]. Weerasekera et al. [53] demonstrated that glucose and sucrose significantly enhance the growth, adhesion, and biofilm formation of *Candida* species. Their study reported maximal planktonic growth at 30 mM of glucose after 14 h of culture, with both 30 mM and 60 mM concentrations of glucose and sucrose eliciting significant increases in adhesion, as indicated by the MTT activity (from 0.17 to >0.019) in both mono- and co-culture conditions. Furthermore, optimal biofilm formation was observed in *Candida albicans* and *Candida tropicalis* with 30 mM of sucrose. The evidence underscores the strong association between quorum sensing (QS), quorum sensing inhibition (QSI), and environmental and nutritional factors, including the type of carbon source available [54]. QS, a pivotal regulatory mechanism in microbial social behavior, plays a key role in biofilm development and interspecies communication. Consistent with prior research, our findings confirm that both glucose and sucrose effectively support the planktonic growth of yeasts. Notably, *Candida* sp. SW4-6 exhibited robust growth in the presence of either glucose or sucrose, suggesting that these sugars are essential carbon sources for yeast metabolism. However, the mechanisms underlying sucrose’s superior effects on growth and nitrogen metabolite removal compared to glucose remain unclear and warrant further investigation.

The activity of microbes in biofilters can be significantly influenced by environmental conditions, both in general applications and specifically in recirculating aquaculture systems [53,54,55]. In a recirculating aquaculture system, the total ammonia-N removal increased six-fold from 6 to 30 °C but decreased significantly at 36 °C [56]. Similarly, in a multi-stage surface flow constructed wetland used for treating polluted river water, temperature affected the expression of the nitrification-related functional gene *nirBD* (nitrite reductase), which influenced the removal efficiency of total nitrogen and nitrate nitrogen. In another study, a water temperature of ≤17 °C resulted in a low removal efficiency for total nitrogen and nitrate-N, while ammonium nitrogen removal decreased when the water temperature was ≤11 °C [57]. In addition to temperature, salinity is another crucial factor influencing nitrogen removal, as it affects the nitrogen metabolism absorption capacity of the microalgae–bacteria consortia [58]. Therefore, it is believed that under optimal conditions, probiotics would present the highest performance in removing nitrogen metabolites from water. In this study, *Candida* sp. SW4-6 had the ability to remove ammonia-N and nitrite-N at the conditions of 0–3.5% NaCl and temperature 20~25 °C. However, more efficient ammonia-N and nitrite-N removal was recorded without NaCl supplementation and at 30 °C, which is due to the better growth conditions of *Candida* sp. SW4-6 (Supplementary Table S4).

## 5. Conclusions

The study concludes that a candidate probiotic capable of effectively degrading nitrite-N was isolated from the shrimp ponds. The ITS sequence and phylogenetic analysis confirmed that the isolate was *Candida* sp., which did not cause any mortality among the tested aquatic animals. The study also found that *Candida* sp. SW4-6 facilitated the degradation of both ammonia-N and nitrite-N under when sucrose was used as a carbon source and at a high C/N ratio. The gene expression of nitrite reductase was positively related to this degradation. Furthermore, under conditions of 0 °C and 30% NaCl, *Candida* sp. SW4-6 demonstrated enhanced capabilities in removing both ammonia-N and nitrite-N. Therefore, the study suggests that *Candida* sp. SW4-6 is a promising probiotic for enhancing the water quality in aquaculture ponds. It is recommended for practical application that the probiotic meets a minimum environmental C/N ratio requirement of 15 and utilizes sucrose as a carbon source to optimize the removal of nitrogen metabolites.

## Figures and Tables

**Table 1:** Virulence assessment and culture environment of aquatic animals after a 7-day challenge with *Candida* sp. SW4-6 through immersion. F: Freshwater; S: Saltwater (35 psu).

Categories	Common Name	Organism	Body Weight (g)	Environment	Mortality (%)
Fish	Malawi hawk cichlid	*Aristochromis christyi*	0.08 ± 0.001	F	0
	Common carp	*Cyprinus carpio*	1.52 ± 0.06	F	0
	Hump-head	*Cyrtocara moorii*	0.11 ± 0.01	F	0
	Milk fish	*Chanos chanos*	1.51 ± 0.06	S	0
	Speckled blue grouper	*Epinephelus cyanopodus*	99.00 ± 20.25	S	0
	Blue-and-yellow grouper	*Epinephelus flavocaeruleus*	112.00 ± 14.57	S	0
	Suckermouth catfish	*Hypostomus plecostomus*	0.53 ± 0.02	F	0
	Tilapia	*Oreochromis* sp.	2.50 ± 0.09	F	0
	Guppy	*Poecilia reticulata*	0.20 ± 0.01	F	0
	Blunthead cichlid	*Tropheus moorii*	2.30 ± 0.13	F	0
Crustaceans	Red claw crayfish	*Cherax quadricarinatus*	0.52 ± 0.03	F	0
	White shrimp	*Penaeus vannamei*	5.27 ± 0.12	S	0
	Tiger shrimp	*Penaeus monodon*	0.03 ± 0.001	S	0
	Spiny lobster	*Panulirus homarus*	26.68 ± 1.87	S	0
Bivalves	Iron clam	*Cyclina sinensis*	33.48 ± 1.24	S	0
	Portuguese oyster	*Crassostrea angulata*	125.15 ± 9.79	S	0

**Table 2:** Tolerance of *Candida* sp. SW4-6 to different temperature conditions and NaCl concentrations. −: no growth; +: colony diameter < 1 mm; ++: colony diameter > 1 mm.

Temperature (°C)																	
10		20		25			28		30		35			40		50	
−		+		++			++		++		++			++		−	
NaCl (%)																	
0.5	1		1.5		2	3		4		5		6	8		10		12
++	++		++		++	++		++		++		++	++		+		+

**Table 3:** Influence of various carbon sources and C/N ratios on ability of *Candida* sp. SW4-6 to reduce nitrite-N. Data are presented as mean ± S.E. Different letters within each carbon source indicate significant differences among various C/N ratios (a, b, c), while within each C/N ratio, different letters indicate significant differences between carbon sources (x, y). The significance level was determined at *p* < 0.05.

Carbon Source	C/N Ratio	Nitrite-N Concentration (mg L^−1^) After Inoculation (h)					
		0	6	12	24	48	72
Glucose	10	5.10 ± 0.02 ^a, x^	4.46 ± 0.11 ^a, y^	4.16 ± 0.06 ^a, y^	2.58 ± 0.10 ^a, x^	2.35 ± 0.04 ^a, x^	2.00 ± 0.07 ^a, x^
	15	5.03 ± 0.17 ^a, x^	4.65 ± 0.09 ^a, x^	4.22 ± 0.05 ^a, y^	1.87 ± 0.15 ^b, x^	1.51 ± 0.17 ^b, x^	1.10 ± 0.16 ^b, x^
	20	5.12 ± 0.05 ^a, x^	4.55 ± 0.22 ^a, x^	4.13 ± 0.06 ^a, y^	0.98 ± 0.19 ^c, x^	0.43 ± 0.09 ^c, x^	0.23 ± 0.08 ^c, x^
Sucrose	10	5.10 ± 0.02 ^a, x^	4.68 ± 0.03 ^a, x^	4.52 ± 0.12 ^a, x^	1.65 ± 0.26 ^a, y^	1.31 ± 0.23 ^a, y^	1.13 ± 0.25 ^a, y^
	15	5.04 ± 0.18 ^a, x^	4.70 ± 0.04 ^a, x^	4.57 ± 0.13 ^a, x^	1.34 ± 0.45 ^a, x^	0.19 ± 0.04 ^b, y^	0.00 ± 0.00 ^b, y^
	20	5.15 ± 0.06 ^a, x^	4.66 ± 0.04 ^a, x^	4.49 ± 0.02 ^a, x^	0.52 ± 0.24 ^b, x^	0.00 ± 0.00 ^c, y^	0.00 ± 0.00 ^b, y^

**Table 4:** Influence of various carbon sources and C/N ratios on ability of *Candida* sp. SW4-6 to reduce ammonia-N. Different letters within each carbon source indicate significant differences among various C/N ratios (a, b, c), while within each C/N ratio, different letters indicate significant differences between carbon sources (x, y). The significance level was determined at *p* < 0.05.

Carbon Sources	C/N Ratios	Ammonia-N Concentration (mg L^−1^) After Inoculation (h)					
		0	6	12	24	48	72
Glucose	10	5.00 ± 0.15 ^a, x^	4.52 ± 0.07 ^a, y^	3.78 ± 0.21 ^a, x^	1.94 ± 0.33 ^a, x^	1.22 ± 0.31 ^a, x^	1.13 ± 0.18 ^a, x^
	15	5.04 ± 0.26 ^a, x^	4.81 ± 0.12 ^a, x^	3.52 ± 0.06 ^ab, y^	0.56 ± 0.17 ^b, x^	0.00 ± 0.00 ^b, x^	0.00 ± 0.00 ^b, x^
	20	4.99 ± 0.19 ^a, x^	4.79 ± 0.36 ^a, x^	3.34 ± 0.16 ^b, y^	0.00 ± 0.00 ^c, x^	0.00 ± 0.00 ^b, x^	0.00 ± 0.00 ^b, x^
Sucrose	10	5.01 ± 0.05 ^a, x^	4.82 ± 0.03 ^a, x^	3.82 ± 0.24 ^a, x^	0.92 ± 0.57 ^a, x^	0.52 ± 0.01 ^a, y^	0.34 ± 0.01 ^a, y^
	15	4.95 ± 0.12 ^a, x^	4.71 ± 0.10 ^a, x^	3.87 ± 0.18 ^a, x^	0.00 ± 0.00 ^b, y^	0.00 ± 0.00 ^b, x^	0.00 ± 0.00 ^b, x^
	20	4.91 ± 0.08 ^a, x^	4.82 ± 0.01 ^a, x^	3.81 ± 0.13 ^a, x^	0.00 ± 0.00 ^b, x^	0.00 ± 0.00 ^b, x^	0.00 ± 0.00 ^b, x^

**Table 5:** Variations in relative gene expression of nitrite reductase in *Candida* sp. SW4-6 across different carbon sources and C/N ratios. Different letters within each carbon source indicate significant differences among various C/N ratios (a, b, c), while within each C/N ratio, different letters indicate significant differences between carbon sources (x, y). The significance level was determined at *p* < 0.05.

Carbon Source	C/N Ratio	Relative Gene Expression of Nitrite Reductase After Inoculation (h)					
		0	6	12	24	48	72
Glucose	10	1.0 ± 0.2 ^a, x^	59.4 ± 15.0 ^b, x^	88.9 ± 17.2 ^c, y^	272.2 ± 38.6 ^b, y^	182.1 ± 37.9 ^c, y^	262.1 ± 13.5 ^b, y^
	15	1.0 ± 0.2 ^a, x^	146.2 ± 8.3 ^a, y^	135.8 ± 11.0 ^b, y^	307.9 ± 12.4 ^b, y^	287.2 ± 27.9 ^b, y^	443.0 ± 46.6 ^a, y^
	20	1.0 ± 0.2 ^a, x^	165.7 ± 23.3 ^a, y^	305.0 ± 10.6 ^a, y^	591.2 ± 59.7 ^a, y^	374.8 ± 7.2 ^a, y^	144.4 ± 15.0 ^c, y^
Sucrose	10	1.0 ± 0.2 ^a, x^	95.7 ± 79.9 ^c, x^	5551.3 ± 35.6 ^b, x^	18414.9 ± 1961.4 ^a, x^	5583.6 ± 1424.3 ^b, x^	8706.7 ± 635.3 ^a, x^
	15	1.0 ± 0.2 ^a, x^	809.6 ± 129.5 ^b, x^	3967.5 ± 800.3 ^b, x^	18379.8 ± 2033.4 ^a, x^	15413.5 ± 856.1 ^a, x^	6695.0 ± 859.4 ^b, x^
	20	1.0 ± 0.2 ^a, x^	1734.2 ± 74.3 ^a, x^	12203.3 ± 1825.8 ^a, x^	14376.0 ± 471.3 ^b, x^	15720.5 ± 917.1 ^a, x^	1366.6 ± 211.9 ^c, x^

**Table 6:** Influence of temperature and NaCl concentration on the nitrite-N reduction by *Candida* sp. SW4-6 using sucrose as the carbon source. Different letters within each NaCl level indicate significant differences among different temperatures (a, b, c), while within each temperature, different letters indicate significant differences between NaCl levels (x, y). The significance level was determined at *p* < 0.05.

NaCl (%)	Temperature (°C)	Nitrite-N Concentration (mg L^−1^) After Inoculation (h)					
		0	6	12	24	48	72
0	20	5.08 ± 0.06 ^a, x^	4.91 ± 0.03 ^a, x^	4.88 ± 0.12 ^a, x^	2.96 ± 0.11 ^a, y^	0.26 ± 0.04 ^a, y^	0.10 ± 0.02 ^a, y^
	25	5.09 ± 0.11 ^a, x^	4.95 ± 0.07 ^a, x^	4.90 ± 0.05 ^a, x^	0.87 ± 0.84 ^b, y^	0.18 ± 0.04 ^a, x^	0.01 ± 0.01 ^b, y^
	30	4.91 ± 0.20 ^a, x^	4.91 ± 0.11 ^a, x^	4.22 ± 0.21 ^b, y^	0.29 ± 0.24 ^b, y^	0.03 ± 0.03 ^b, x^	0.00 ± 0.00 ^b, x^
3.5	20	5.13 ± 0.05 ^a, x^	4.91 ± 0.08 ^a, x^	4.75 ± 0.18 ^a, x^	4.85 ± 0.11 ^a, x^	0.89 ± 0.16 ^a, x^	0.21 ± 0.02 ^a, x^
	25	5.10 ± 0.09 ^a, x^	4.91 ± 0.08 ^a, x^	4.78 ± 0.10 ^a, x^	3.77 ± 0.55 ^b, x^	0.21 ± 0.05 ^b, x^	0.11 ± 0.04 ^b, x^
	30	4.90 ± 0.06 ^a, x^	4.86 ± 0.05 ^a, x^	4.70 ± 0.08 ^a, x^	2.57 ± 0.16 ^c, x^	0.08 ± 0.14 ^b, x^	0.00 ± 0.00 ^c, x^

**Table 7:** Influence of temperature and NaCl concentration on the ammonia-N reduction caused by *Candida* sp. SW4-6 using sucrose as the carbon source. Different letters within each NaCl level indicate significant differences among different temperatures (a, b, c), while within each temperature, different letters indicate significant differences between NaCl levels (x, y, z). The significance level was determined at *p* < 0.05.

NaCl (%)	Temperature (℃)	Ammonia-N Concentration (mg L^−1^) After Inoculation (h)					
		0	6	12	24	48	72
0	20	5.08 ± 0.14 ^a, x^	4.57 ± 0.24 ^a, x^	4.00 ± 0.09 ^a, y^	0.00 ± 0.00 ^a, y^	0.00 ± 0.00 ^a, x^	0.00 ± 0.00 ^a, x^
	25	5.10 ± 0.20 ^a, x^	4.27 ± 0.17 ^a, x^	2.72 ± 0.21 ^b, y^	0.00 ± 0.00 ^a, x^	0.00 ± 0.00 ^a, x^	0.00 ± 0.00 ^a, x^
	30	5.12 ± 0.07 ^a, x^	3.80 ± 0.09 ^b, y^	0.04 ± 0.07 ^c, y^	0.00 ± 0.00 ^a, x^	0.00 ± 0.00 ^a, x^	0.00 ± 0.00 ^a, x^
35	20	5.10 ± 0.27 ^a, x^	4.75 ± 0.33 ^a, x^	4.44 ± 0.26 ^a, x^	2.25 ± 0.33 ^a, x^	0.00 ± 0.00 ^a, x^	0.00 ± 0.00 ^a, x^
	25	5.07 ± 0.25 ^a, x^	4.32 ± 0.12 ^b, x^	3.98 ± 0.12 ^b, x^	0.37 ± 0.39 ^b, x^	0.00 ± 0.00 ^a, x^	0.00 ± 0.00 ^a, x^
	30	5.09 ± 0.15 ^a, x^	4.08 ± 0.05 ^b, x^	2.91 ± 0.21 ^c, x^	0.00 ± 0.00 ^b, x^	0.00 ± 0.00 ^a, x^	0.00 ± 0.00 ^a, x^

## Data Availability

The data presented in this study are available in the article.

## Supplementary Materials

The following supporting information can be downloaded at: https://www.mdpi.com/article/10.3390/microorganisms13010042/s1, Figure S1: Colonial morphology of *Candida* sp. SW4-6; Table S1: Components in N mediums with different C/N ratios used to assess the reduction of nitrite-N and ammonia-N in water; Table S2: Multi-way ANOVA table for nitrite-N reduction by *Candida* sp. SW4-6 under different salinities, temperatures and time points; Table S3: Multi-way ANOVA table for ammonia-N reduction by *Candida* sp. SW4-6 under different salinities, temperatures and time points; Table S4: Growth of *Candida* sp. SW4-6 at different levels of NaCl and temperature. Different letters within each NaCl level indicate significant differences among different temperature (a, b, c), while within each temperature, different letters indicate significant differences between NaCl levels (x, y, z). The significance level (*p* value) is set at 0.05; Table S5: Virulence assessment and culture environment of aquatic animals after a 7-day challenge with *Candida* sp. SW4-6 through injection at a dosage of 10^9^ cfu (g body weight)^−1^. F: Freshwater; S: Saltwater (35 psu).
