# Supplementary material for: Exploring the Potential of Candida sp. SW4-6 as a Probiotic for Enhancing Water Quality in Aquaculture
Source: Microorganisms. 2024 Dec 29;13(1):42. doi: 10.3390/microorganisms13010042 (PMC11768024; doi:10.3390/microorganisms13010042)
Supplement: Supplementary file 1 [file microorganisms-13-00042-s001.zip › microorganisms-3406754-supplementary.pdf]

Table S1. Components in N mediums with different C/N ratios used to assess the reduction of nitrite-N and ammonia-N in water.

Components	C/N ratio		
	**10**	**15**	**20**
Glucose / Sucrose (g L^{-1})	0.1250 / 0.1191	0.1875 / 0.1786	0.2500 / 0.2381
KH_2PO_4 (g L^{-1})	2	2	2
$MgSO_4 \cdot 7H_2O$ (g L^{-1})	0.05	0.05	0.05
$FeSO_4 \cdot 7H_2O$ (g L^{-1})	0.02	0.02	0.02
NaCl (g L^{-1})	35	35	35

Table S2. Multi-way ANOVA table for nitrite-N reduction by *Candida* sp. SW4-6 under different salinities, temperatures and time points.

	**SS**	**DF**	**MS**	**F**	***p* value**
Salinity	5.237	1	5.237	127.127	<0.001
Temperature	6.389	2	3.194	77.536	<0.001
Time	433.396	5	86.679	2103.977	<0.001
Salinity * Temperature	0.011	2	0.005	0.133	0.875
Salinity * Time	19.504	5	3.901	94.687	<0.001
Temperature * Time	13.367	10	1.337	32.447	<0.001
Salinity * Temperature * Time	1.418	10	0.142	3.443	<0.001
Error	2.760	67	0.041		
Total	1456.717	103			

Table S3. Multi-way ANOVA table for ammonia-N reduction by *Candida* sp. SW4-6 under different salinities, temperatures and time points.

	SS	DF	MS	F	*p* value
Salinity	4.725	1	4.725	197.099	<0.001
Temperature	10.459	2	5.230	218.147	<0.001
Time	473.225	5	94.645	3947.961	<0.001
Salinity * Temperature	0.366	2	0.183	7.638	0.001
Salinity * Time	8.969	5	1.794	74.825	<0.001
Temperature * Time	19.253	10	1.925	80.311	<0.001
Salinity * Temperature * Time	8.758	10	0.876	36.533	<0.001
Error	1.726	72	0.024		
Total	1020.924	108			

Table S4. Growth of *Candida* sp. SW4-6 at different levels of NaCl and temperature. Different letters within each NaCl level indicate significant differences among different temperature (a, b, c), while within each temperature, different letters indicate significant differences between NaCl levels (x, y, z). The significance level (P value) is set at 0.05.

NaCl (%)	Temperature (°C)	Bacterial counts ($\times 10^4$ CFU mL^{-1}) at various culture time (h)					
		0	6	12	24	48	72
0	20	2.3 ± 0.3 ^{a,x}	5.7 ± 1.0 ^{c,x}	22.0 ± 6.0 ^{b,x}	173.3 ± 115.5 ^{b,x}	1240.0 ± 28.3 ^{a,x}	1140.0 ± 28.3 ^{a,x}
	25	2.3 ± 0.2 ^{a,x}	13.5 ± 4.1 ^{b,x}	38.7 ± 2.3 ^{b,x}	833.3 ± 377.5 ^{b,x}	1280.0 ± 203.0 ^{a,x}	1340.7 ± 131.1 ^{a,x}
	30	2.5 ± 0.6 ^{a,x}	23.6 ± 3.3 ^{a,x}	170.0 ± 31.4 ^{a,x}	1640.0 ± 521.2 ^{a,x}	1326.7 ± 201.3 ^{a,x}	1520.0 ± 622.3 ^{a,x}
3.5	20	2.1 ± 0.3 ^{a,x}	1.7 ± 0.4 ^{b,y}	4.0 ± 2.0 ^{b,y}	133.3 ± 92.4 ^{a,x}	840.0 ± 28.3 ^{a,y}	520.0 ± 141.4 ^{b,y}
	25	1.8 ± 0.2 ^{a,x}	3.8 ± 2.4 ^{ab,y}	14.7 ± 8.1 ^{b,y}	266.7 ± 30.6 ^{a,x}	870.0 ± 14.1 ^{a,x}	660.0 ± 242.5 ^{b,y}
	30	1.9 ± 0.6 ^{a,x}	16.0 ± 11.0 ^{a,x}	33.3 ± 11.4 ^{a,y}	526.7 ± 375.4 ^{a,y}	1253.3 ± 623.6 ^{a,x}	1073.3 ± 83.3 ^{a,x}

Table S5. Virulence assessment and culture environment of aquatic animals after a 7-day challenge with *Candida* sp. SW4-6 through injection at a dosage of 10^9 CFU (g body weight)$^{-1}$. F: Freshwater; S: Saltwater (35 psu).

Categories	Common name	Organism	Body weight (g)	Environment	Mortality (%)
Fish	Malawi hawk cichlid	*Aristochromis christyi*	0.09 ± 0.001	F	0
	Common carp	*Cyprinus carpio*	1.76 ± 0.05	F	0
	Hump-head	*Cyrtocara moorii*	0.11 ± 0.02	F	0
	Milk fish	*Chanos chanos*	1.61 ± 0.07	S	0
	Speckled blue grouper	*Epinephelus cyanopodus*	100.12 ± 24.13	S	0
	Blue-and-yellow grouper	*Epinephelus flavocaeruleus*	103.08 ± 23.33	S	0
	Suckermouth catfish	*Hypostomus plecostomus*	0.43 ± 0.01	F	0
	Tilapia	*Oreochromis* sp.,	1.98 ± 0.04	F	0
	Blunthead cichlid	*Tropheus moorii*	2.20 ± 0.14	F	0
Crustaceans	Red claw crayfish	*Cherax quadricarinatus*	0.61 ± 0.05	F	0
	White shrimp	*Penaeus vannamei*	4.89 ± 0.22	S	0
	Tiger shrimp	*Penaeus monodon*	0.11 ± 0.001	S	0

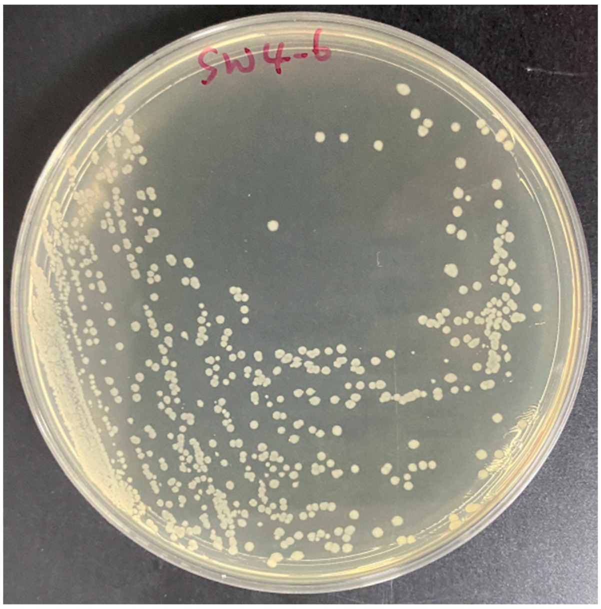

Supplementary Figure S1. Colonial morphology of *Candida* sp. SW4-6.
